# Supplementary material for: Inhibition of UVB-Induced Inflammation by Laminaria japonica Extract via Regulation of nc886-PKR Pathway
Source: Nutrients. 2020 Jun 30;12(7):1958. doi: 10.3390/nu12071958 (PMC7400497; doi:10.3390/nu12071958)
Supplement: Supplementary file 1 [file nutrients-12-01958-s001.pdf]

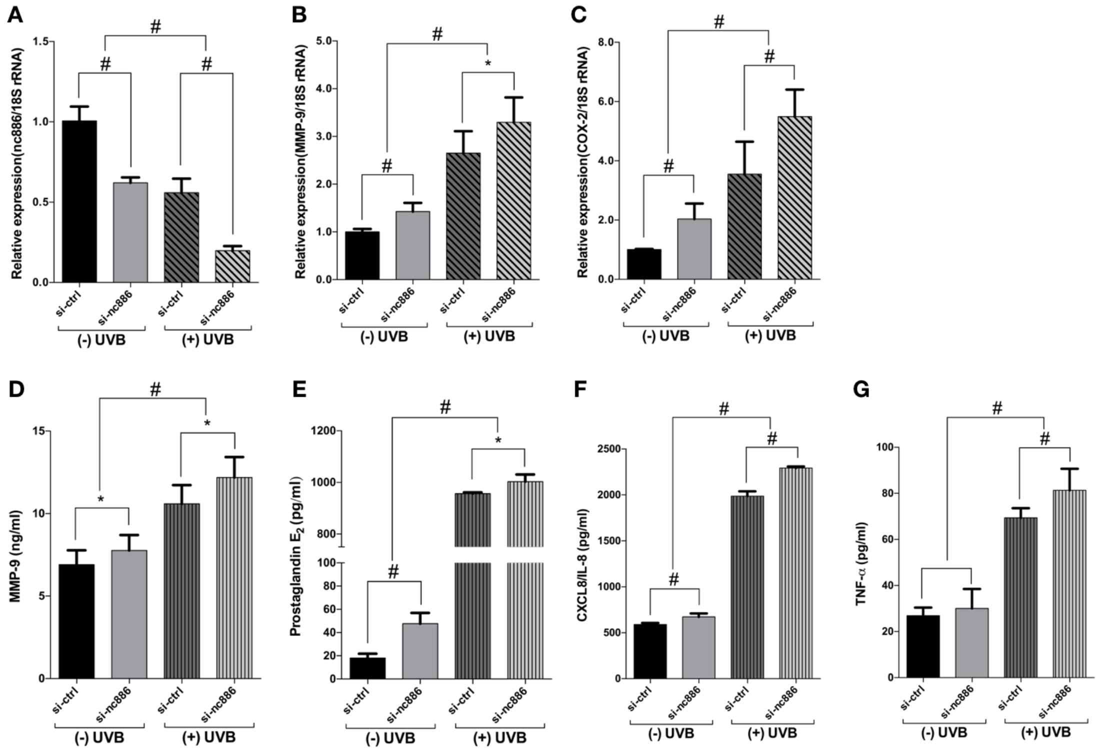

Figure.S1. Knockdown of nc886 further increases the expression of MMP-9 and COX-2, which are increased by UVB. UVB irradiation (5mJ/cm^2) was administered 24 h after transfection with 250 pmol of each anti-oligonucleotide. Each of the RNA samples was analyzed with quantitative real-time polymerase chain reaction (qRT-PCR) and the cell culture fluid was subjected to ELISA. Each of the factors (A) nc886, (B) MMP-9, (C) COX-2 was indicated by a fold change compared with the control. The mRNA level was normalized with the expression of 18S rRNA. The values of (D) MMP-9, (E) PGE_2, (F) IL-8, and (G) TNF-α were indicated by their absolute concentrations. **P < 0.05, #P < 0.01*, compared with basal levels.
